# MiR-323a-3p acts as a tumor suppressor by suppressing FMR1 and predicts better esophageal squamous cell carcinoma outcome

**DOI:** 10.1186/s12935-022-02541-x

**Published:** 2022-03-29

**Authors:** Yu Men, Yirui Zhai, Lihong Wu, Lipin Liu, Wenjue Zhang, Wei Jiang, Nan Bi, Yongmei Song, Zhouguang Hui, Luhua Wang

**Affiliations:** 1 Department of VIP Medical Services & Radiation Oncology, National Cancer Center/National Clinical Research Center for Cancer/Cancer Hospital, Chinese Academy of Medical Sciences and Peking Union Medical College, Beijing, China; 2 Department of Radiation Oncology, National Cancer Center/National Clinical Research Center for Cancer/Cancer Hospital, Chinese Academy of Medical Sciences and Peking Union Medical College, Beijing, China; 3 Genecast Precision Medicine Technology Institute, Beijing, China; 4 Department of Radiation Oncology, Beijing Hospital, National Center of Gerontology, Institute of Geriatric Medicine, Chinese Academy of Medical Sciences, Beijing, China; 5 Department of Radiation Oncology, National Cancer Center/National Clinical Research Center for Cancer/Cancer Hospital & Shenzhen Hospital, Chinese Academy of Medical Sciences and Peking Union Medical College, Beijing, Guangdong China; 6 The State Key Laboratory of Molecular Oncology, National Cancer Center/National Clinical Research Center for Cancer/Cancer Hospital, Chinese Academy of Medical Sciences and Peking Union Medical College, Beijing, China

**Keywords:** Esophageal squamous cell carcinoma, miR-323a-3p, FMR1

## Abstract

**Background:**

Esophageal squamous cell carcinoma (ESCC) has unfavorable outcomes with the highest incidence seen in China. Accordingly, exploring effective molecular biomarkers is of great value. MicroRNAs (miRNAs) are posttranscriptional regulators of gene expression and modulate numerous biological processes in tumors. Our study aimed to identify prognostic miRNAs and investigate their role in ESCC.

**Methods:**

Prognosis-related plasma miRNAs were detected by miRNA microarray and qRT-PCR. Functional assays and molecular mechanism studies were used to investigate the role of miRNA in ESCC.

**Results:**

Over-expression of miR-323a-3p was associated with a favorable prognosis. MiR-323a-3p negatively regulated proliferation, migration, and invasion. Through biological predictions, the fragile X mental retardation 1 (FMR1) was found to be a potential target of miR-323a-3p. Further investigation revealed that miR-323a-3p directly targeted and suppressed FMR1. MiR-323a-3p and FMR1 mRNA, as well as miR-323a-3p and the FMR1-encoded protein FMRP, showed negative correlations. Luciferase activity of FMR1-3′-UTR, but not mutant counterparts, was decreased by mimic compared with that of the control. The compromised cell proliferation, migration, and invasion induced by transfection with miR-323a-3p mimic were rescued by transfection with a FMR1 expression plasmid. Tumors induced by miR-323a-3p overexpressed ESCC cells grew significantly slower in vivo and resulted in smaller tumor masses. Metastatic lung colonization was also inhibited by miR-323a-3p overexpression.

**Conclusions:**

MiR-323a-3p was significantly associated with survival and acted as a tumor suppressor by inhibiting proliferation, migration, and invasion via the regulation of FMR1. MiR-323a-3p is a promising biomarker and may be a potential therapeutic target.

**Supplementary Information:**

The online version contains supplementary material available at 10.1186/s12935-022-02541-x.

## Background

Esophageal cancer is the seventh most common and sixth most fatal cancer worldwide with approximately 52% of the cases occurring in China [1]. Esophageal squamous cell carcinoma (ESCC) is specifically highly prevalent in China, and accounts for 95% of all esophageal cancer cases. Compare with esophageal adenocarcinoma, which is most common in Western countries, ESCC has many differences in aspects of etiology, epidemiology, clinical course, and even responsiveness to treatments. In addition, due to the high invasiveness and early metastasis of ESCC, patients are usually diagnosed with locally advanced disease and a poor prognosis with 5-year overall survival (OS) of 20–34% [2, 3]. In view of the particularity and suboptimal survival of patients with esophageal cancer in China, specific explorative studies regarding prognostic predictors are needed in order to choose proper individual treatments.

MicroRNAs (miRNAs) are small noncoding RNAs of 18–22 nucleotides in length that bind to the 3′-untranslated regions (UTRs) of target mRNAs to post-transcriptionally regulate gene expression, mainly through gene silencing and inhibition of translation [4]. Accordingly, miRNAs have emerged as a new class of biomolecules with important roles in cellular functions and are regarded as potential biomarkers and valuable regulators of many physiological and pathological processes [5]. Emerging evidence has demonstrated that miRNAs play important roles in tumor processes and their aberrant expression is closely associated with tumor proliferation, differentiation, apoptosis, and metastasis [5]. Aberrant expression of miRNAs is also closely related to clinical outcomes in several types of carcinomas, including lung cancer [6], breast cancer [7], and liver cancer [8]. However, compared to these cancers, an understanding regarding the roles of miRNAs in ESCC is lacking.

To identify miRNAs involved in ESCC carcinogenesis, we first performed miRNA microarray analyses to survey miRNA expression in plasma specimens of 12 patients with ESCC. The aberrant expression was further confirmed using quantitative real-time polymerase chain reaction (qRT-PCR) analysis of plasma specimens from 30 patients with ESCC. We discovered that low expression of miR-323a-3p was significantly associated with poor prognosis which suggested that miR-323a-3p might be a tumor suppressor. Through literature review, we found that miR-323a-3p was related to several cancers [9–11]. Like Chen et al. [11] showed that miR-323a-3p suppressed the growth of osteosarcoma cells. Overexpression of miR-323a-3p decreased the cell viability, colon formation and induced the apoptosis of osteosarcoma cells. Additionally, down-regulation of miR-323a-3p was significantly correlated with the poor survival outcome of the bladder cancer patients and miR-323a-3p was frequently downregulated in bladder cancer tissues and three cell lines [10]. These results also suggested the potential tumor suppressive role of miR-323a-3p in cancers. Thus, next it seems very important to clarify the function and mechanism of miR-323a-3p in ESCC. Furthermore, we determined the tumor suppressive function of miR-323a-3p was achieved by targeting the fragile X mental retardation 1 (FMR1) gene to inhibit proliferation, migration, and invasion, both in vitro and in vivo.

## Methods

### Patients and plasma samples

We collected plasma samples from 30 patients with locally advanced stage ESCC who received radiotherapy or chemoradiotherapy at the Cancer Hospital, Chinese Academy of Medical Sciences from 2007 to 2010. None of the patients had undergone surgery. Eligibility criteria were as follows: age between 18 and 70 years, Eastern Cooperative Oncology Group (ECOG) performance status (PS) ≤ 1 and no history of other malignances. All patients provided written informed consent and the study was approved by the Institutional Review Boards of Cancer Hospital, Chinese Academy of Medical Sciences.

### MiRNAs isolation

Total RNA, which included the miRNA fraction, was extracted from serum samples using the mirVana PARIS Kit (Ambion, USA) and from cultured cells using Trizol reagent (Invitrogen) according to the manufacturers’ instructions. The purity and concentration of all RNA samples were evaluated according to their 260/280 nm absorbance ratios, which were determined using a NanoDrop 2000C spectrophotometer (NanoDrop Technologies, Rockland, DE, USA).

### MiRNAs microarray and qRT-PCR analyses

Expression levels of the miRNAs were determined using an AB TaqMan Human MicroRNA Array (Applied Biosystems), which included probes for 748 mature human miRNA sequences. Expression of miR-323a-3p was determined by the stem-loop qRT-PCR method. Briefly, complementary DNA (cDNA) was prepared using 2 µg total RNA as template and synthesized using a SuperScript™ First-Strand Synthesis System for RT-PCR Kit (Invitrogen, USA) based on the specific stem-loop RT primers for miR-323a-3p or U6 (RiboBio, China). U6 small nuclear RNA was used as an internal control for miR-323a-3p. For quantification of FMR1 mRNA, total RNA was reverse transcribed into cDNA using a First-Strand cDNA Synthesis Kit (Promega, USA) using FMR1-specific primers. The FMR1 primer sequences were as follows: forward, 5′-CGGCAAATGTGTGCCAAAGA-3′; reverse, 5′-ATGTGCTCGCTTTGAGGTGA-3′. The qRT-PCR was performed on an ABI Prizm 7300 Sequence Detection System (Applied Biosystems) using Light Cycler DNA Master SYBR Green I Mix (Roche, Switzerland) according to the manufacturer’s instructions. All qRT-PCR reactions were performed in triplicate. The expression levels of miR-323a-3p and FMR1 mRNA were quantified using the 2^−ΔΔCt^ method and normalized to internal control levels.

### Bioinformatic prediction of miRNA target genes

The predicted target genes of miR-323a-3p were investigated using the iterative algorithms of TargetScan (http://www.targetscan.org/), miRbase (http://microrna.sanger.ac.uk/), and miRDB (http://mirdb.org/).

### Cell culture

Seven ESCC cell lines, KYSE-30, KYSE-140, KYSE-150, KYSE-180, KYSE-450, KYSE-510, and YES-2, were cultured in RPMI-1640 medium (Gibco BRL, USA) supplemented with 10% fetal bovine serum (FBS; Gibco BRL, USA) at 37 °C in humidified air containing 5% carbon dioxide. Throughout the experiments, the cells were used in the logarithmic phase of growth.

### Cell transfection

We selected KYSE-30, KYSE-150, and YES-2 cells for the functional studies. Synthesized miR-323a-3p mimics, inhibitor, mimic negative control (NC), and NC inhibitor were purchased from RiboBio (Guangzhou, China). Cell transfections were performed at a concentration of 50 nM using Lipofectamine 2000 (Invitrogen, USA) according to the manufacturer’s protocol. Total RNA and protein were harvested 48 h after transfection and used for qRT-PCR and western blot analysis, respectively, as described.

### Cell proliferation, migration, and invasion assays

Cell proliferation analysis was performed using transfected cells. The cells were seeded into 96-well plates at approximately 3 × 10^3^ cells per well in 200 μL of medium. At 0, 24, 48, and 72 h post seeding, 100 μL 5% MTS reagent (Promega, USA) in phosphate-buffered saline (PBS) was added to each well and incubated for 2 h. Absorbance was then read at a wavelength of 490 nm using a microplate reader (Bio-Rad, USA).

Cell migration and invasion assays were analyzed using Transwell chambers (Costar, USA) and Matrigel matrix (BD Biosciences, USA). Briefly, 1 × 10^5^ transfected cells in 200 μL serum-free medium were seeded into the upper chamber with 100 μL of 2% Matrigel for migration or without Matrigel for invasion assays. The lower chambers were filled with 600 μL RPMI-1640 containing 20% FBS. The chambers were then incubated at 37 °C with 5% CO_2_ for 8–20 h. The cells that migrated or invaded the membrane of the insert were fixed, strained, and analyzed. Cells in 10 random microscopic fields (100× magnification) were counted for each insert.

### Western blot analysis

Cells were lysed with cold lysis buffer consisting of 1% NP-40 supplemented with a complete protease inhibitor tablet (Sigma, USA) for 30 min on ice. Equivalent amounts of total-protein extracts were separated by 10% SDS-PAGE and transferred to polyvinylidene difluoride (PVDF) membranes (Millipore, USA). The blots were blocked with 3% bovine serum albumin (BSA) for 1 h and the incubated with primary antibodies against fragile X mental retardation protein (FMRP, 1:500; Proteintech, China) and β-actin (control, 1:5000, Santa Cruz, USA) at 4 °C overnight. After washing with PBS three times, the membranes were incubated with their corresponding secondary antibodies at room temperature for 1 h. The membranes were washed again and then incubated with the chemiluminescence substrate. Photographs were taken using an ImageReader LAS-4000 (Fujifilm, Japan) and analyzed using Image-Pro Plus 6.0 software.

### Plasmid construction and luciferase activity assays

The FMR1 3′-UTR target site sequence and sequence with an eight-nucleotide mutation in the miR-323a-3p target site were synthesized and cloned downstream of the luciferase gene in the pEGFP-C1 luciferase vector (Generay, China). The resulting vectors were named FMR1-3′-UTR-WT and FMR1-3′-UTR-Mut, respectively. Cells were seeded into 12-well plates and co-transfected with the constructed plasmids, internal control Renilla luciferase plasmid (pRL-SV40), and mimic or mimic NC using Lipofectamine 2000 (Invitrogen, USA). After 48 h, cells were harvested and luciferase activity measured using a Dual Luciferase Assay Kit (Promega, USA) and a Synergy H1 hybrid reader (Biotek, USA). Results were normalized to Renilla luciferase activity and the data expressed as relative luciferase activity. Experiments were performed in triplicate on three separate occasions.

### Retroviral infection

The lentivirus for miR-323a-3p was purchased from GeneChem. The miR-323a-3p lentivirus was prepared and used to transfect cells according to the manufacturer’s protocol.

### Analysis of in vivo tumorigenicity

BALB/c nu/nu male mice, 4 weeks old, were purchased from Vital River for the in vivo tumorigenicity study. All experimental procedures were approved by the Institutional Animal Welfare Guidelines. Mice were injected subcutaneously in the hind legs with 2 × 10^6^ cells in 0.2 mL. The size of the tumors was determined based on caliper measurements of subcutaneous tumor masses. Tumor volumes were calculated according to the formula 4/3πr_1_ × r_2_^2^ (r_1_ > r_2_). Each experimental group included six mice and the experiments were repeated three times. In addition, mice were injected via tail veins with 1 × 10^6^ cells in 0.2 mL. Mice were killed by cervical dislocation.

### Statistical analysis

Differences between groups were estimated using the Student’s t test and the Chi-square test. OS was defined as the time between the date of diagnosis and death. Local recurrence-free survival (LRFS) was defined as the time between the date of diagnosis to local or regional lymph node recurrence, last follow-up, or death. Survival data were analyzed using the Kaplan–Meier method and compared with the log-rank test. All experiments were performed at least three times. For the expression of miR-323a-3p, relative qualification (RQ) > 1 was regarded as overexpression and RQ ≥ 2 or ≤ 0.5 were considered significantly different. Data are expressed as mean ± SEM. Differences between groups were analyzed by two-tailed Student’s t-test. Statistical significance is indicated by *P < 0.05, **P < 0.01, and ***P < 0.001 versus the relevant control. The data were analyzed using SPSS 19.0 software or Prism 7.0 software (GraphPad).

## Results

### Upregulation of miR-323a-3p correlated with good ESCC clinical outcome

**Fig. 1:**
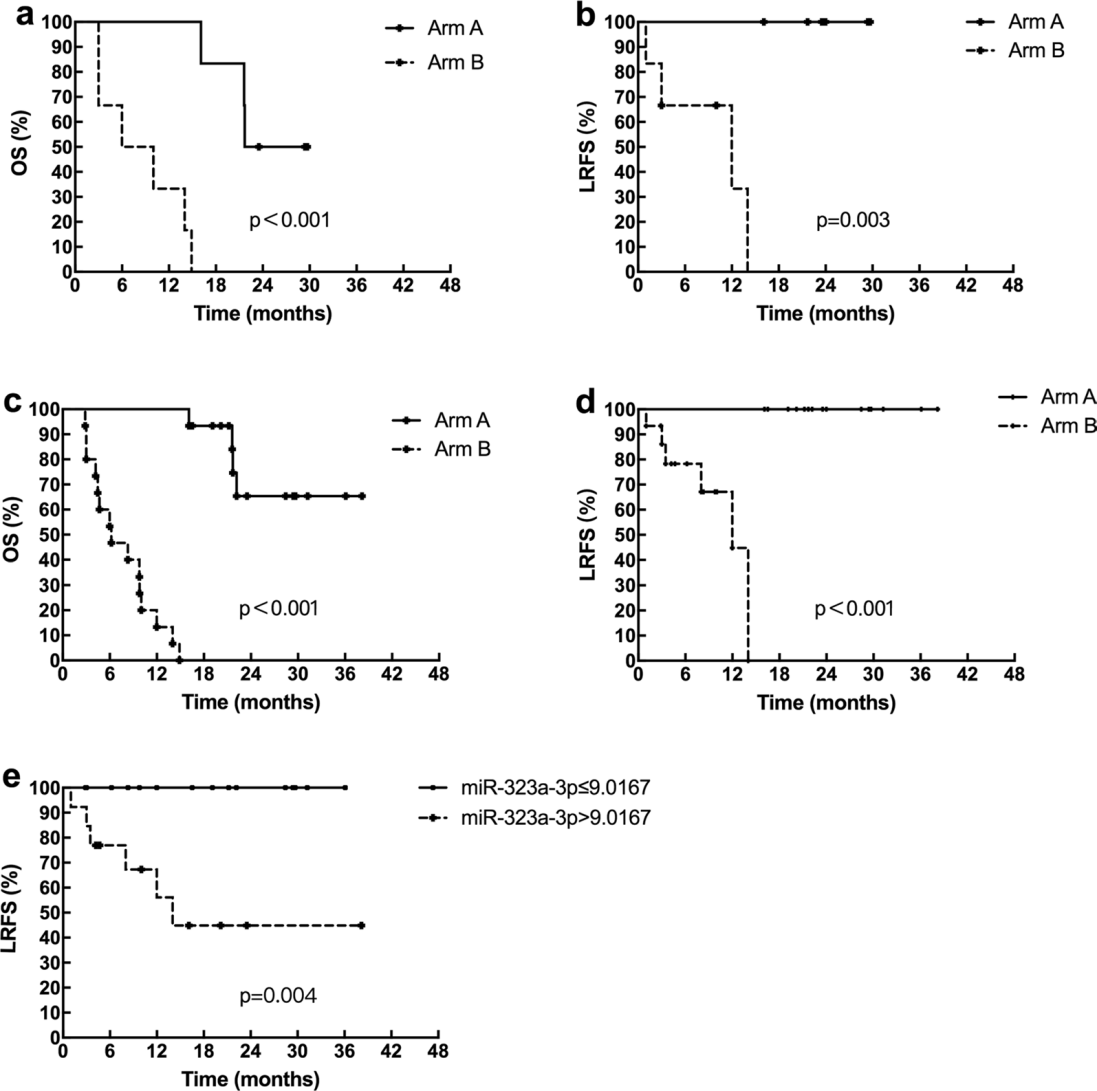
Comparison of survival between the different groups. **a** OS of 12 patients; **b** LRFS of 12 patients; **c** OS of 30 patients; **d** LRFS of 30 patients; **e** LRFS between low (> 9.0167) and high (≤ 9.0167) expression of miR-323a-3p

We selected 12 patients and divided them into two groups according to the difference in prognosis, Arm A and Arm B. The clinical characteristics for the two groups were well balanced (Table 1). Survival of patients in Arm A was significantly better than that of patients in Arm B. The median OS in Arm A was not reached and in Arm B was 10 months (p < 0.001; Fig. 1a). The median LRFS was not reached in Arm A and was 6.5 months in Arm B (p = 0.003; Fig. 1b). Using miRNA microarray, miR-323a-3p was detected in the plasma of the two groups and found to be differentially expressed. The miR-323a-3p was upregulated in Arm A (RQ = 2.15), which was the group with good prognosis. (The other differentially expressed miRNAs were shown in Additional file 1).

**Table 1:** Characteristics of patients

Characteristics	Total (n = 12)			Total (n = 30)		
	Arm A	Arm B	P	Arm A	Arm B	P
Gender						
Male/female	6/0	5/1	0.296	13/2	14/1	0.543
Age, years						
Median	65	65	0.558	65	65	0.477
Tumor location						
Up/middle/low	2/3/1	3/2/1	0.354	5/9/1	4/11/0	0.519
Stage						
II/III/IVA	1/3/2	1/4/1	0.788	3/8/4	2/9/4	0.879
Treatments						
RT^a^/CRT^b^/RT + TT^c^	3/2/1	3/2/1	1.000	7/6/2	6/8/1	0.706
OS, months						
Median	NR^d^	10	< 0.001	NR	6.2	< 0.001
LRFS, months						
Median	NR	6.5	0.003	NR	6.2	< 0.001

^a^Radiotherapy

^b^Concurrent chemoradiotherapy

^c^Target therapy

^d^Not reached

To confirm the microarray findings, qRT-PCR was performed using plasma from 30 patients with ESCC. These patients were also divided into two groups according to the differences in prognosis (Fig. 1c, d). The clinical characteristics were also well balanced between the two groups (Table 1). The qRT-PCR results showed that miR-323a-3p was overexpressed in the group with good clinical outcomes (RQ = 2.1). The median value of miR-323a-3p expression in the 30 samples was defined as the cutoff value to classify low expression of miR-323a-3p (> 9.0167) and high expression of miR-323a-3p (≤ 9.0167). Downregulation of miR-323a-3p expression was found to be significantly related to a poor LRFS (p = 0.004; Fig. 1e).

### MiR-323a-3p inhibited cell proliferation, migration, and invasion in vitro

**Fig. 2:**
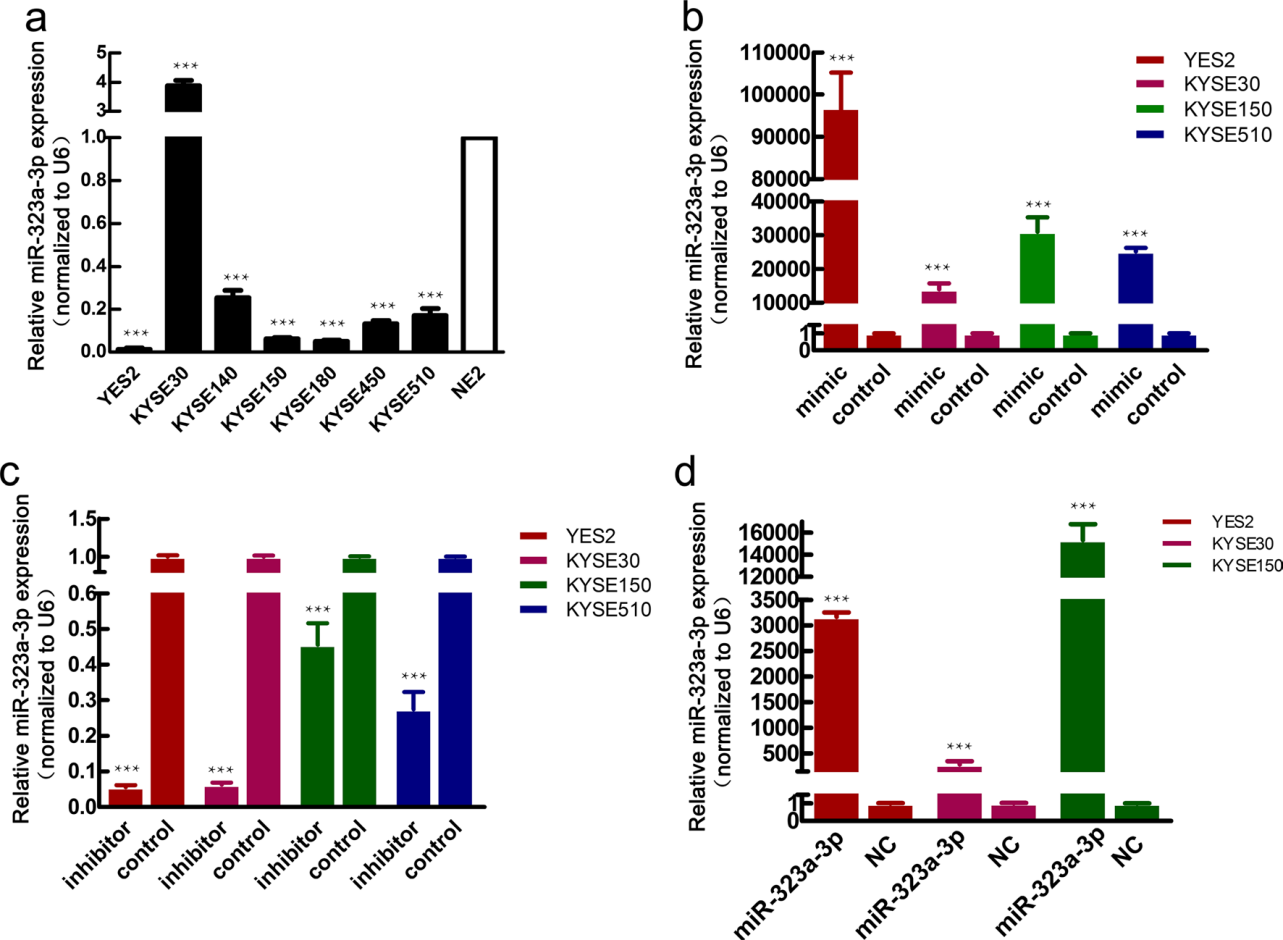
Expression levels of miR-323a-3p in different ESCC cells. **a** Expression levels of miR-323a-3p in HEEC-NE2 and ESCC cells; **b** MiR-323a-3p was overexpressed by the miR-323a-3p mimic in ESCC cells; **c** MiR-323a-3p was knocked down by the miR-323a-3p inhibitor in ESCC cells; **d** MiR-323a-3p was overexpressed by lentivirus infection in ESCC cells (MOI = 3). The data are representative of three independent experiments. The expression levels of miR-323a-3p were quantified using the 2−ΔΔCt method and normalized to internal control levels. *p < 0.05, **p < 0.01, and ***p < 0.001

Seven ESCC cell lines and human esophageal epithelial cell (HEEC)-NE2 cells were evaluated by qRT-PCR for miR-323a-3p expression levels. In most ESCC cell lines, except KYSE-30, the expression of miR-323a-3p was significantly lower than that in the NE2 cells (Fig. 2a). This suggested a tumor-suppressive role for miR-323a-3p in ESCC.

To explore the functions of miR-323a-3p in ESCC, we transfected ESCC cell lines with miR-323a-3p mimics or mimic NC and inhibitor or inhibitor NC. As shown by the transfection efficiencies in Fig. 2b, c, miR-323a-3p expression levels were significantly increased by miR-323a-3p mimic and decreased by miR-323a-3p inhibitor, respectively.

**Fig. 3:**
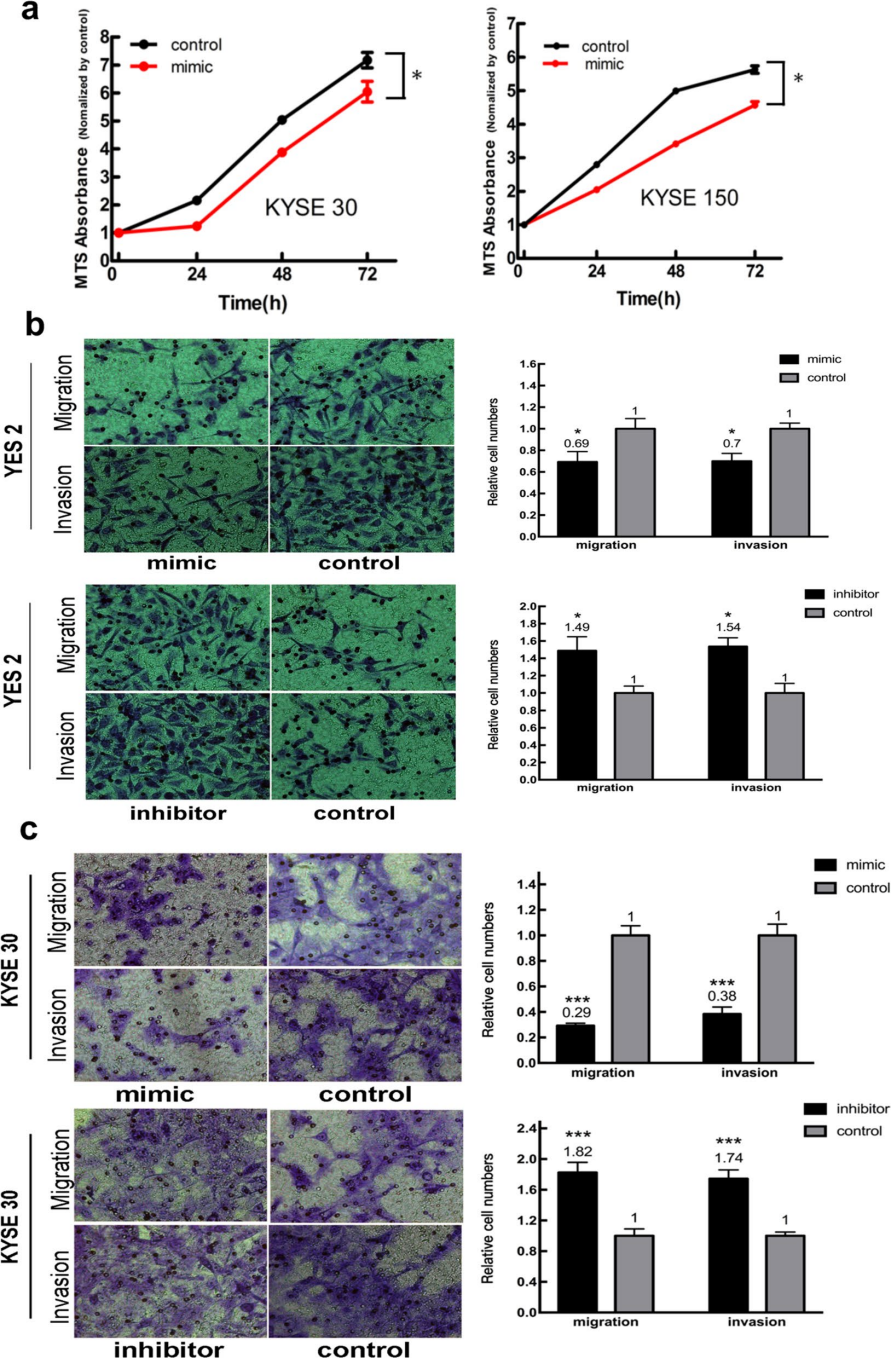
Effects between the expression levels of miR-323a-3p and the biological behaviors of ESCC cells. **a** Cell proliferation abilities were suppressed in KYSE 30 and KYSE 150 cells with miR-323a-3p overexpression compared to those in negative control (NC) transfected cells; **b** cell migration and invasion abilities were determined in YES 2 cells with enforced or reduced miR-323a-3p expression compared to those in NC transfected cells; **c** cell migration and invasion abilities were determined in KYSE 30 cells with enforced or reduced miR-323a-3p expression compared to those in NC transfected cells. *p < 0.05, **p < 0.01, and ***p < 0.001

Next, we examined the effects of miR-323a-3p on cellular functions. Data from MTS assays were used to generate growth curves for the various KYSE-30 and KYSE-150 transfected cells. We determined the upregulation of miR-323a-3p resulted in significantly decreased cell proliferation compared with that of NC (Fig. 3a). We also counted the number of cells that migrated to the basal side of membranes in Transwell assays. The results revealed that migration and invasion capabilities were increased when miR-323a-3p expression was silenced. In contrast, both these capabilities were decreased by treatment with the miR-323a-3p mimic (Fig. 3b, c). These results suggested that miR-323a-3p was able to inhibit cell proliferation, migration, and invasion in vitro.

### FMR1 was a direct target of miR-323a-3p

**Fig. 4:**
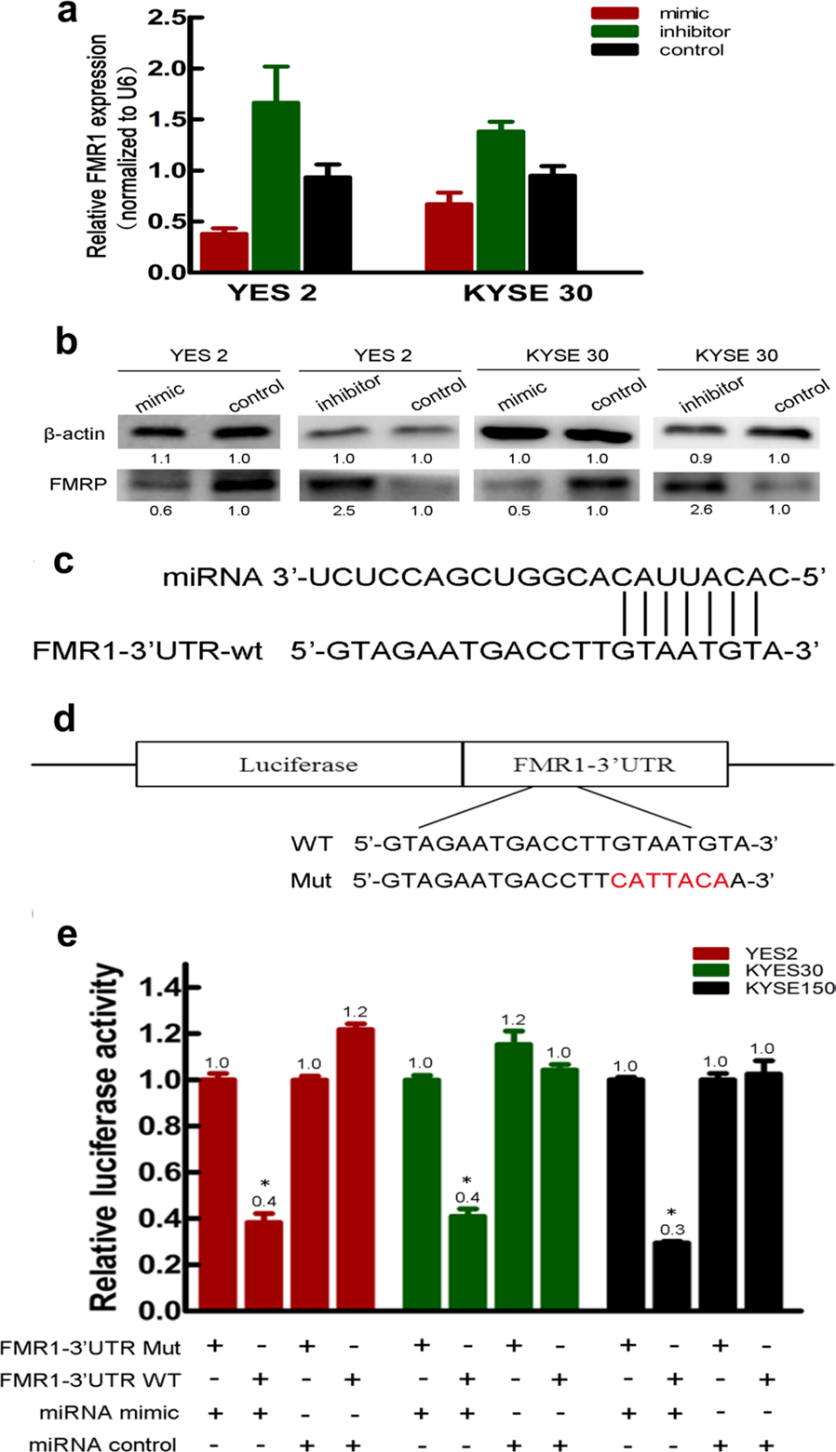
FMR1 is the target gene of miR-323a-3p. **a** The negative correlation between expression levels of miR-323a-3p and FMR1 mRNA; **b** the negative correlation between expression levels of miR-323a-3p and FMRP; **c** MiR-323a-3p can directly targeted the 3′-UTR of FMR1; **d** FMR1 3′-UTR-WT and FMR1 3′-UTR-MUT; **e** the relationship between miR-323a-3p and FMR1 is approved by Luciferase-reporter assay. *p < 0.05

To further investigate the mechanisms by which miR-323a-3p acted as a tumor suppressor, we searched TargetScan, miRbase, and MIRDB and ultimately identified FMR1 as a potential target of miR-323a-3p. To determine whether FMR1 mRNA expression negatively correlated with miR-323a-3p expression in ESCC cells, both YES-2 and KYSE-30 cells were analyzed using qRT-PCR. The results indicated that overexpression of miR-323a-3p in ESCC cells resulted in downregulation of FMR1 mRNA compared with controls, while knockdown of miR-323a-3p resulted in upregulation of FMR1 mRNA (Fig. 4a). The relationship between miR-323a-3p expression and that of FMR1-encoded protein FMRP was also evaluated. Western blot analysis revealed an inverse relationship between the two. Specifically, FMRP expression was markedly decreased in miR-323a-3p mimic-transfected ESCC cells, but increased in miR-323a-3p inhibitor-transfected ESCC cells (Fig. 4b). It was concluded that miR-323a-3p repressed FMR1 at both the mRNA and protein level.

To further investigate whether miR-323a-3p directly targeted the 3′-UTR of FMR1 as predicted (Fig. 4c), we performed luciferase reporter assays. Wild-type or mutant 3′-UTR of FMR1 was inserted downstream of a firefly luciferase reporter gene to create FMR1 3′-UTR-WT and FMR1 3′-UTR-MUT, respectively (Fig. 4d). Each firefly luciferase vector and renilla luciferase vector (pRL-SV40) were co-transfected with mimic or mimic NC into KYSE-30 cells. A significant decrease in relative luciferase activity of approximately 60–70% was observed in ESCC cells transfected with FMR1 3′-UTR-WT and miR-323a-3p mimic compared to those transfected with the mimic NC. However, the luciferase activity of ESCC cells transfected with the mutant FMR1 reporter did not change (Fig. 4e). These results suggested that miR-323a-3p could bind directly to the 3′-UTR of FMR1.

### MiR-323a-3p regulated cell functions through FMR1

**Fig. 5:**
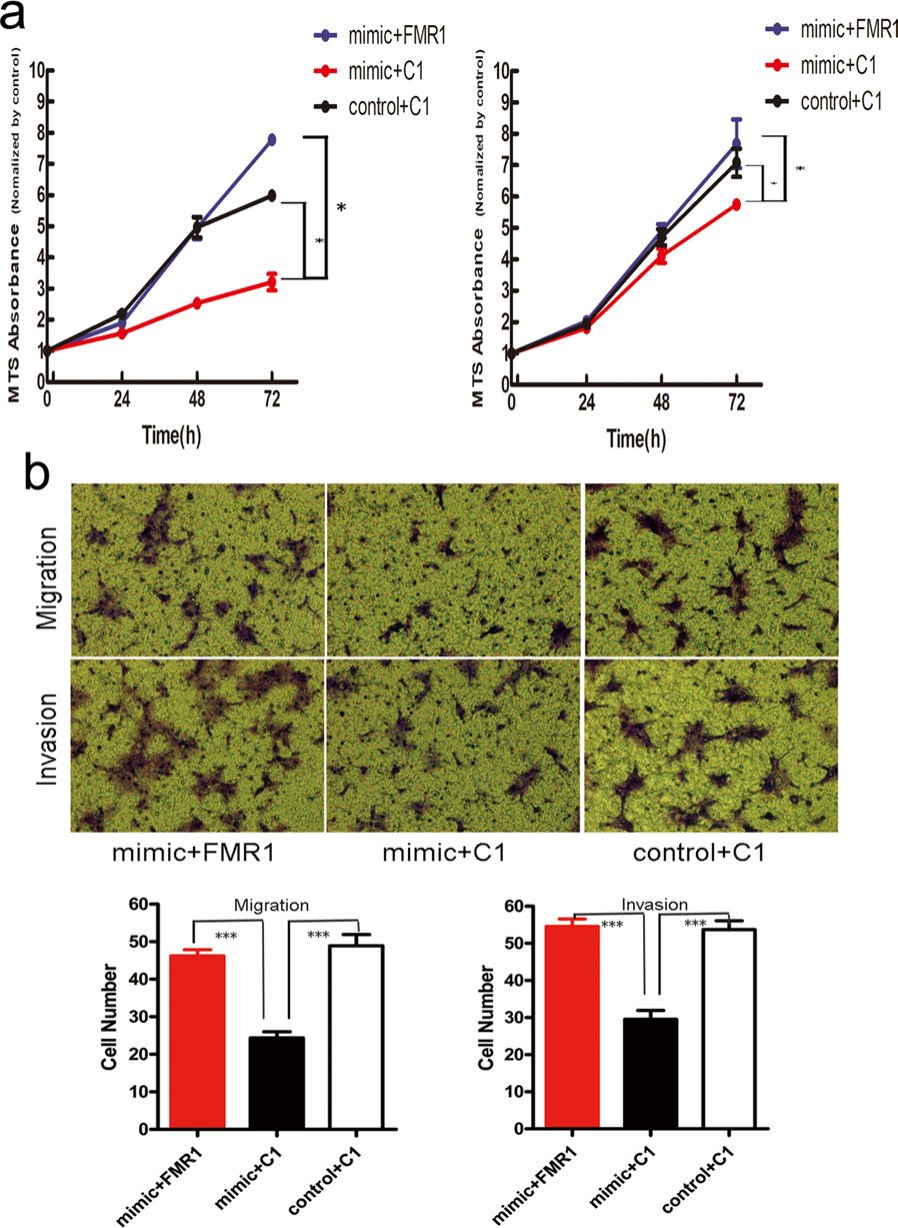
Forced expression of FMR1 abolishes the phenotype created by miR-323a-3p mimic transfection. **a** Cell proliferation ability was determined in ESCC cells transfected with miR-323a-3p mimic plus a control vector (C1), NC plus C1 and miR-323a-3p mimic plus FMR1 expression plasmid; **b** cell migration and invasion abilities were determined in ESCC cells transfected with miR-323a-3p mimic plus C1, NC plus C1 and miR-323a-3p mimic plus FMR1 expression plasmid. *p < 0.05, ***p < 0.001

To investigate the contribution of FMR1 to the biological functions of miR-323a-3p regarding proliferation, migration, and invasion, we examined whether reconstitution of FMR1 had an effect on miR-323a-3p-induced cell function changes. We co-transfected an expression vector carrying the FMR1 open reading frame (ORF) without the 3′-UTR or a control vector (C1) together with the miR-323a-3p mimic. Cell proliferation, migration, and invasion abilities were all rescued by the restored expression of FMR1 in the miR-323a-3p mimic-treated cells (Fig. 5a, b). Taken together, these results demonstrated the miR-323a-3p-mediated cell function changes in ESCC cells were at least partially caused by repression of FMR1.

### MiR-323a-3p suppressed tumor growth and metastasis in vivo

To assess the effects of miR-323a-3p on tumor repression in vivo, two pooled sets of ESCC cell lines were stably transduced with miR-323a-3p lentiviral vectors or negative controls. As expected, leti-miR-323a-3p-infected ESCC cells expressed a much higher level of miR-323a-3p than that of the control-transduced cells (Fig. 2d).

**Fig. 6:**
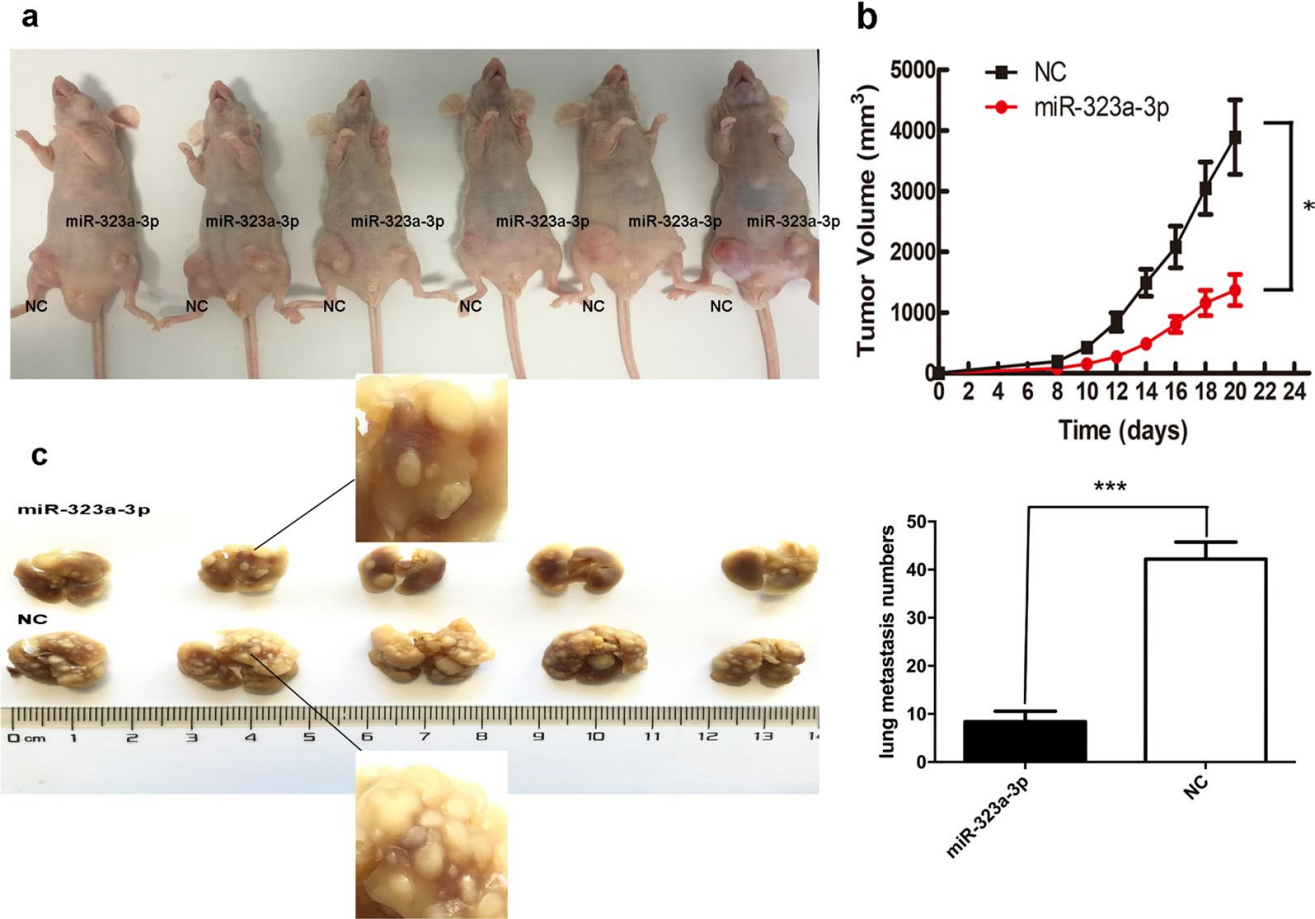
MiR-323a-3p’s effect on tumor growth and metastasis in vivo. **a** The representative tumor masses. Mice were injected subcutaneously with overexpression miR-323a-3p ESCC cells into the left leg and the control ESCC cells into right leg; **b** growth of both ESCC-miR-323a-3p and ESCC-control cells-induced tumor in nude mice was monitored weekly; **c** the representative lung metastasis of each group (in miR-323a-3p group, the first line in the picture, from left to right, the number of the lung metastasis was 14, 13, 3, 7 and 5, respectively; in NC group, it was 42, 52, 44, 43 and 30, respectively.). *p < 0.05, ***p < 0.001

Transduced KYSE-30 cells were chosen for subsequent studies. Both KYSE-30 cell pools were implanted subcutaneously into 6 mice for each group and tumor growth was monitored weekly. Tumors in the lenti-miR-323a-3p group grew significantly slower than those of the negative control group, resulting in smaller tumor masses (Fig. 6a, b). To further confirm the correlation between miR-323a-3p expression and tumor metastasis, both cell pools were separately injected into the tail veins of mice. After 3 months, the animals were analyzed for lung metastases. All mice in each group exhibited lung metastases; however, the numbers of lung metastases in the lenti-miR-323a-3p group were markedly lower than those in the control group (Fig. 6c). These results indicated that miR-323a-3p inhibited tumor growth and metastasis in vivo.

## Discussion

Esophageal cancer is one of the most aggressive cancers worldwide and a leading cause of cancer-related deaths [12, 13]. Squamous cell carcinoma (SCC) and adenocarcinoma (AC) are the two major histologic subtypes of esophageal cancer and their incidence varies among geographic areas [14]. Approximately 52% of world-wide esophageal cancers occur in China and ESCC accounts for approximately 95% of all those cases [1]. ESCC is clinically challenging, not only to treat, but also with respect to choosing efficient treatments. Accordingly, there is an urgent need for the proper predictors.

MiRNAs are critical regulators of transcriptional and post-transcriptional gene silencing, which are involved in multiple developmental processes of cancers. The role of miRNAs in ESCC progression is becoming better recognized and there is increasing interest in identifying the key miRNAs involved in aggressive ESCC phenotypes in order to improve diagnosis, predict prognosis, and develop new therapeutic strategies [15–25]. Thus, we hypothesized that miRNAs might play an important role in the prediction of prognosis for ESCC and may be helpful in decision making for individual treatment options. In our current study, we found that some miRNAs expressed differently between the 2 arms (Additional file 1), the miR-323a-3p was one of the miRNAs. And elevated levels of miR-323a-3p in plasma were closely associated with better outcomes. Furthermore, we performed a series of experiments to determine the functions of miR-323a-3p in ESCC cells, which showed that enhancing miR-323a-3p expression suppressed cell proliferation, migration, and invasion in vitro and suppressed tumor growth and metastasis behaviors in vivo. Furthermore, enhancing the expression of miR-323a-3p inhibited each of these aggressive phenotypes by targeting FMR1. Taken together, these data indicate that miR-323a-3p served as a tumor suppressor in ESCC. Our study is the first to demonstrate the role of miR-323a-3p in ESCC, as well as to identify the target gene.

MiR-323a-3p exists in a miRNA cluster in the chromosomal region 14q32.31, which is a region critical for placental growth and embryonic development. This suggests that miR-323a-3p may be useful in the early detection of ectopic pregnancy (EP) [26]. In addition, evidence suggests that miR-323a-3p plays important roles in several diseases, such as rheumatoid arthritis and Alzheimer’s disease [19, 27, 28], and may be a potential therapeutic biomarker for these diseases. Otherwise, miR-323-3p is a candidate for diagnosing cardiomyopathy in Friedreich’s ataxia patients [29] and a candidate biomarker for coronary artery disease in acute coronary syndrome patients [30]. However, there are few studies regarding the role of miR-323a-3p in cancer, which have focused on pancreatic ductal adenocarcinoma (PDAC) [31], bladder cancer [10], osteosarcoma [11], cervical cancer [32], and glioblastoma [33], not ESCC. As mentioned before, in PDAC, bladder cancer and osteosarcoma, miR-323a-3p is a tumor suppressor, whereas in cervical cancer and glioblastoma it is a tumor promoter. Importantly, this suggests that miR-323a-3p may play different roles and exhibit different antitumor effects when it comes to carcinogenesis of different types of cancer. Our data showed for ESCC that high expression of miR-323a-3p was related to better LRFS, inhibited cell proliferation, migration, and invasion in vitro, and suppressed tumor growth and metastasis in vivo. These results indicate that miR-323a-3p acted as an anti-tumor factor and could be predictive of a better prognosis in ESCC. Moreover, it was previously demonstrated that overexpression of miR-323a-3p increased the activity of a Wnt reporter and that the Wnt/β-catenin pathway is important for radiosensitivity [27, 28]. Considering the patients in our study all received radiotherapy, we propose that miR-323a-3p was a potential factor for radiosensitivity, which should be further explored.

Dysregulation of miRNAs has been shown to contribute to tumor initiation and progression by regulating target genes [5]. After using the iterative algorithms to predict target genes of miR-323a-3p, it showed that AFT6, KRAS, EED and FMR1 were all the potential target gene. In our current study, we demonstrated that FMR1 was the target gene of miR-323a-3p in ESCC. An inverse correlation was identified between the expression of miR-323a-3p and levels of FMR1 mRNA or FMRP and the biological functions of miR-323a-3p were determined by modulating FMR1. Yi et al. [34] also predicted and confirmed that miR-323a-3p directly binds to the 3′-UTR of FMR1, which is in agreement with our results. Based on the literature, FMR1 is definitely related to fragile X syndrome (FXS), which is an X-linked disorder [35]; however, few studies have focused on cancers. Patients with FXS are known to have a low risk of malignant cancers [36], but Kalkunte et al. [37] reported a case report of a boy with FXS developing a glioblastoma. However, the glioblastoma in this boy behaved as a cytogenetically or physically unusual tumor compared with that of glioblastomas in adult patients, perhaps due to the absence of FMRP. This indicates that FMRP has aggressive functions when it comes to cancers. Liu et al. [38] evaluated the genetic background of hepatocellular carcinoma (HCC) and investigated differential expression of genes. They validated that FMR1 is one of the genes upregulated in HCC. In addition, FMRP and FMR1 mRNA levels correlate with prognostic indicators of aggressive breast cancer, lung metastases probability, and triple negative breast cancer (TNBC) [39]. These results further demonstrate the cancer-promoting activity of FMR1 and the anti-tumor activity of miR-323a-3p. Furthermore, Luca et al*.* [39] demonstrated that FMRP binds mRNAs involved in epithelial mesenchymal transition (EMT). Thus, according to the functional biological effects of miR-323a-3p, especially regarding changes in migration and invasion, we hypothesized that EMT might also be a process that is influenced by the expression of miR-323a-3p and FMR1. This is reasonable as EMT has been shown in many cancers to promote cancer migration and intravasation from primary cancer during the metastatic cascade. It is this process by which epithelial cells lose their characteristic polarity and adopt a mesenchymal phenotype.

## Conclusions

In summary, miR-323a-3p acted as a tumor-suppressive miRNA in ESCC. It was predictive of a better prognosis and may be helpful in deciding proper effective treatment choices. Further analysis showed that miR-323a-3p mediated tumor-related functions by targeting FMR1. To our knowledge, this study provides the first evidence to identify miR-323a-3p as a promising biomarker for ESCC.

## Supplementary Information

**Additional file 1: Table S1.** The differentially expressed miRNAs by microarray.

## Data Availability

The datasets during and/or analyzed during the current study are available from the corresponding author on reasonable request.

## Abbreviations

3′-UTR: 3′-Untranslated regions
AC: Adenocarcinoma
AD: Alzheimer’s disease
BSA: Bovine serum albumin
ECOG: Eastern cooperative oncology group
EMT: Epithelial mesenchymal transition
EP: Ectopic pregnancy
ESCC: Esophageal squamous cell carcinoma
FMR1: FMRP translational regulator 1
FMRP: Fragile-X mental retardation protein
FXS: Fragile X syndrome
HCC: Hepatocellular carcinoma
HEEC: Human esophageal epithelial cell
LRFS: Local recurrence free survival
miRNAs: MicroRNAs
MTS: Cell Titer 96 AQueous One Solution Cell Proliferation Assay
MUT: Mutant
NC: Negative control
ORF: Open reading frame
OS: Overall survival
PBS: Phosphate-buffered saline
PDAC: Pancreatic ductal adenocarcinoma
PS: Performances status
PVDF: Polyvinylidene difluoride
qRT-PCR: Quantitative real time polymerase chain reaction
RA: Rheumatoid arthritis
RQ: Relative qualification
SCC: Squamous cell carcinoma
TNBC: Triple negative breast cancer
WT: Wildtype
