# Supplementary material for: MiR-323a-3p acts as a tumor suppressor by suppressing FMR1 and predicts better esophageal squamous cell carcinoma outcome
Source: Cancer Cell Int. 2022 Mar 29;22:140. doi: 10.1186/s12935-022-02541-x (PMC8966287; doi:10.1186/s12935-022-02541-x)
Supplement: Supplementary file 1 — Additional file 1: Table S1. The differentially expressed miRNAs by microarray. [file 12935_2022_2541_MOESM1_ESM.docx]

Additional file 1

Table S1：The differentially expressed miRNAs by microarray

|  | **RQ >2** | **RQ<0.5** |
| --- | --- | --- |
|  | **Assay** | **Assay** |
| 1 | hsa-miR-30e-3p-000422 | hsa-miR-3p-4380948 |
| 2 | hsa-miR-340-4395369 | hsa-miR-106b-4373155 |
| 3 | hsa-miR-367-4373034 | hsa-miR-218-4373081 |
| 4 | hsa-miR-574-3p-4395460 | hsa-miR-618-4380996 |
| 5 | hsa-miR-302b-4378071 | hsa-miR-20b-4373263 |
| 6 | hsa-miR-296-5p-4373066 | hsa-miR-518d-3p-4373248 |
| 7 | hsa-miR-520d-5p-4395504 | hsa-miR-645-001597 |
| 8 | hsa-miR-323-3p-4395338 | hsa-miR-222-4395387 |
| 9 | hsa-miR-151-3p-002254 | hsa-miR-19b-4373098 |
| 10 | hsa-miR-454-4395434 | hsa-miR-515-3p-4395480 |
| 11 | hsa-miR-126#-000451 | hsa-miR-548d-3p-4381008 |
| 12 | hsa-miR-432-001026 | hsa-miR-140-5p-4373374 |
| 13 | hsa-miR-4373132 | hsa-miR-532-3p-4395466 |
| 14 | hsa-miR-186-4395396 | hsa-miR-4373028 |
| 15 | hsa-miR-30b-4373290 | hsa-miR-328-4373049 |
| 16 | hsa-miR-545#-002266 |  |
